# Extended Lower Blepharoplasty: How Much Skin Can We Resect?

**DOI:** 10.1007/s00266-025-05405-7

**Published:** 2025-11-06

**Authors:** Mariachiara Fabbri, Marta Mariani, Vittoria Murone, Pietro Luciano Serra, Chiara Botti, Giovanni Botti

**Affiliations:** 1 Residency Program in Plastic Surgery, Università Cattolica del “Sacro Cuore”, Largo A. Gemelli 8, 00168 Rome, Italy; 2 Residency Program in Plastic Surgery, Azienda Ospedaliera Universitaria Federico II di Napoli, Via Sergio Pansini 5, 80131 Napoli, Italy; 3 Residency Program in Plastic Surgery, Plastic Surgery Unit, Department of Medical Surgical and Experimental Sciences, University of Sassari, Sassari University Hospital Trust, 07100 Sassari, Italy; 4 Plastic Surgeon in Private Practice at Villa Bella Clinic, Via Europa 55, 25087 Salò, Italy

**Keywords:** Blepharoplasty, Lower blepharoplasty, Festoons, Malar bags, Oculoplasty, Facial rejuvenation

## Abstract

**Introduction:**

Lower eyelid blepharoplasty is a widely performed procedure to address periorbital aging. While traditional techniques primarily focus on the lower eyelid, extended lower blepharoplasty provides a more comprehensive approach by incorporating midface rejuvenation. This study evaluates the safety and efficacy of extended lower blepharoplasty, with a particular focus on its ability to allow the excision of large amounts of excess skin while maintaining eyelid stability and minimizing complications. Consequently, we selected cases in which the primary concern was skin elastosis, characterized by significant skin redundancy and sagging. In contrast, patients whose midfacial aging was predominantly due to progressive adipose or bony atrophy were excluded from the study.

**Methods:**

A retrospective analysis was conducted on patients who underwent extended lower blepharoplasty over a 10 year period (2013–2023). Surgical technique involved a transcutaneous approach with subcutaneous and sub-orbicularis dissection, meticulous tissue repositioning, and deep and superficial canthoplasty for structural support. The study assessed the amount of skin excised and the incidence of postoperative complications, including scleral show and ectropion.

**Results:**

Among the patients included, the amount of skin excised ranged from 6 to 16 mm, with an average of 10 mm. The extended technique demonstrated a low complication rate, with satisfactory aesthetic and functional outcomes in most patients.

**Conclusion:**

Extended lower blepharoplasty is a safe and effective technique for addressing extensive lower eyelid skin laxity and midface aging. The ability to excise substantial amounts of skin while maintaining eyelid stability makes it a valuable alternative to traditional approaches.

**Level of Evidence III:**

This journal requires that authors assign a level of evidence to each article. For a full description of these Evidence-Based Medicine ratings, please refer to the Table of Contents or the online Instructions to Authors www.springer.com/00266.

**Supplementary Information:**

The online version contains supplementary material available at 10.1007/s00266-025-05405-7.

## Introduction

The periorbital region, including the eyelids, plays a pivotal role in facial expression and emotional communication. With advancing age, an increasing number of patients seek consultation for lower eyelid blepharoplasty due to concerns such as skin and orbicularis laxity, malar bags, festoons, or “circles” under the eyes. [1] A youthful appearance of the eyelid–cheek complex is characterized by a single convex line observed on profile view [2], yet age-related alterations can lead to pseudo-herniation of orbital fat and subsequent double-convex lower eyelid contour. The midface may likewise descend, leading to inferiorly displaced cheek fat, skin and orbicularis muscle and, consequently, festoons, malar bags and tear trough deformity onset [3].

Lower eyelid blepharoplasty, a well-established surgical procedure, offers satisfactory cosmetic outcomes and effectively addresses age-related changes in the palpebral and periorbital region, contributing to a more youthful facial appearance.

Currently, lower eyelid blepharoplasty is performed using either the transcutaneous or transconjunctival approach, each with its own set of advantages and drawbacks [4–6].

The transconjunctival approach is typically employed when the primary concern is the removal of fat pads with or without a minimal amount of excess skin. In contrast, the transcutaneous approach is preferred for cases involving a significant amount of excess skin to be excised and usually involves an ample subcutaneous dissection combined with a deep, static or dynamic canthopexy [7] and an anchorage of the orbicularis oculi muscle to the periosteum of the orbital rim (“superficial canthopexy”) [8].

To have a more complete armamentarium capable of correcting defects in both the eyelid and periorbital regions, a third procedure must be added to the two basic techniques of lower blepharoplasty. Actually, while traditional lower blepharoplasty primarily targets the lower eyelid region to address issues such as excess skin, fat protrusion and muscle laxity, extended lower blepharoplasty encompasses a broader scope by incorporating steps to rejuvenate the midface as well.

Extended lower blepharoplasty, particularly beneficial for patients with advanced signs of aging or seeking comprehensive rejuvenation of the lower eyelid–cheek complex, involves extending the dissection of the skin–muscle flap, used in standard lower eyelid blepharoplasty, below the inferior orbital rim. This technique is particularly useful in managing festoons and malar bags, observed in up to 10% of blepharoplasty candidates. The achievement of satisfactory results hinges on the proper suspension of the skin–muscle flap to the lateral canthal periosteum. [9]

The first description of extended blepharoplasty was by Small in 1981. Small's extended blepharoplasty involved dissection of the lower eyelid myo-cutaneous flap beyond the inferior orbital rim and onto the anterior maxilla, aimed at correcting large cheek festoons. [10]

The choice between limited “traditional” and extended approaches depends on factors such as the patient's aesthetic goals, anatomical considerations and the extent of aging changes present in both the lower eyelid and midface regions.

However, determining the appropriate amount of skin to excise with both techniques is crucial to avoid complications such as ectropion, scleral show, eyelid retraction and overall unnatural appearance.

A review of the literature reveals that the amount of skin excision can vary based on individual patient factors such as age, skin elasticity, degree of lower eyelid laxity and desired aesthetic outcomes. [11]

In terms of specific measurements, there is some variation depending on the individual patient's anatomy and the surgical technique employed. However, most surgeons suggest limiting the amount of skin excision to approximately 2 to 4 millimeters. This conservative approach helps maintain adequate support and tension in the lower eyelid to prevent adverse outcomes. [12]

Other studies [13, 14] propose more aggressive approaches to skin excision, particularly in patients with significant lower eyelid laxity or redundant skin, which can be associated with other techniques such as orbicularis muscle suspension and canthoplasty, both static and dynamic, to provide additional support and stability to the lower eyelid, minimizing the risk of complications.

The aim of this article is to present our experience with extended lower blepharoplasty through which we safely and effectively excise large amounts of skin, up to 16 millimeters, while still achieving favorable aesthetic outcomes and minimizing the risk of postoperative complications. The study evaluated the occurrence of postoperative complications such as scleral show or ectropion, and the necessity for a secondary corrective procedure.

## Materials and Methods

This retrospective study analyzes a cohort of patients who underwent extended lower blepharoplasty between 2013 and 2023. Over this ten-year period, a total of 913 lower blepharoplasty procedures were performed, with an average of 90 surgeries per year. Among these, 10% (approximately 10 procedures per year) were classified as extended lower blepharoplasties, totaling 93 cases over the decade. This study specifically focuses on these extended lower blepharoplasty cases.

The primary indications for extended lower blepharoplasty included, besides orbital fat pad herniation, festoons, malar bags, severe lower eyelid and periorbital skin laxity, and infrapalpebral depression. As a retrospective study, patient selection was based on the presence of clinical indications for extended lower blepharoplasty. Formal inclusion and exclusion criteria were not applied. Patient-specific factors such as previous surgery, smoking status and coexisting conditions like exophthalmos were evaluated on an individual basis during the preoperative assessment. These factors did not serve as absolute exclusion criteria but guided the personalized surgical approach to optimize outcomes.

All surgical procedures were performed either by the first or the second senior author at their private clinic, always using the same standardized technique, with both surgeons present during each operation. Postoperative follow-up and photographic documentation were carried out exclusively by the same senior author to ensure consistency and minimize bias. The study adheres to the principle of the Declaration of Helsinki. All patients were elicited regarding the proposed technique, and informed consents were collected.

### Surgical Technique

#### Anesthetic Preparation and Infiltration

General anesthesia or deep sedation was administered to all patients to ensure optimal comfort and safety during the procedure. The surgery was performed under sterile conditions with full monitoring and anesthesiological support. A local anesthetic (mepivacaine 2%) with epinephrine 1:100.000 is then infiltrated into the surgical area.

#### Incision, Flap Dissection and Removal of Herniated Fat Pads

A transcutaneous incision is made in the lower pretarsal skin approximately 2–3 mm from the ciliary margin and extended laterally by 5–10 mm. A skin flap is then elevated, dissecting at a subcutaneous level over approximately 1 cm below the incision. This double layer dissection will facilitate excision of excess skin at the end of surgery. Subsequently, an incision is made in the orbicularis oculi muscle approximately 2 mm from the inferior border of the tarsus, starting below the lateral extremity of the skin incision. A muscle flap is carefully dissected downward to the orbital rim, creating a preseptal plane. Then, the undermining is extended almost in the same plane, between the orbicularis muscle and the SOOF (suborbicularis oculi fat) for about 1 cm. Care must be taken to avoid damaging the endings of the infraorbital nerve, while the superficial fibers of the zygomatico-facial, another sensory nerve located more laterally, do not pose particular problems due to its numerous anastomoses. The orbital septum is then incised, exposing the herniated fat pads, which are either excised or repositioned based on the patient’s specific anatomical needs.

#### Orbicularis Muscle Repositioning and Canthoplasty

Following meticulous hemostasis, the orbicularis muscle flap is vertically redraped over the septum until it lies naturally along the lash line. Lifting the orbicularis also lifts the skin that remains attached to it in the extraorbital portion of the dissection. A canthoplasty is subsequently performed, either static or dynamic [8], depending on the patient’s anatomical characteristics and functional requirements. Static canthoplasty is used to reinforce the eyelid suspension system. In 78% of cases, this was achieved by plicating the canthal ligament using 5/0 Vicryl sutures (Ethicon®), without altering the position of the lateral canthus. In the remaining 22% of cases, where lateral canthal ptosis was present, a canthal repositioning was performed by releasing the lateral canthal tendon and ligament and anchoring the residual stump to the periosteum of the inner orbital rim with 5/0 Nylon sutures (Ethicon®). Dynamic canthoplasty, on the other hand, is indicated when there is a need to lift the lateral canthus, such as when it has moved caudally (canthal dystopia), as frequently seen in cases of ectropion, scleral show, or eyelid bowing. In such cases, bone fixation may be performed using a 1.0 mm surgical drill to create one or two holes in the lateral orbital rim. Through these, 2–4 sutures of 5/0 Nylon (Ethicon®) are passed to secure the lateral tarsus to the periosteum. Canthal symmetry is maintained by referencing the original insertion point and repositioning both sides cranially by an equal amount. In practice, adequate correction is achieved when the lower eyelid margin covers the limbus bilaterally by approximately 2 mm. [7] At this point, the orbicularis muscle excess is excised, and in the remaining muscle a flap is sculped that is then anchored to the superolateral orbital rim through a para-canthal tunnel using a suspending suture (Adamson’s technique) [15, 16]. For this purpose, usually 3 sutures are applied: two fixing the muscle to the orbital rim periosteum, the third between the muscle flap and the deep temporalis fascia, passing through the superficial one.

#### Skin Excision and Closure

**Fig. 1:**
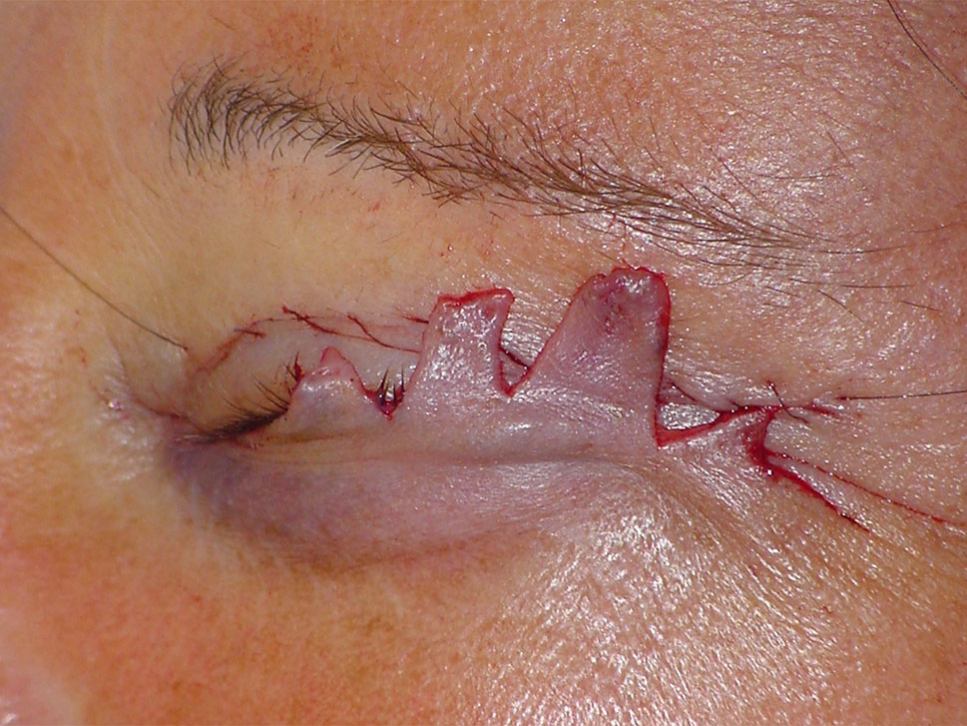
Skin excision leaving 2 mm of excess skin in order to avoid eyelid marginal displacement

The skin flap is then carefully repositioned over the previously stabilized orbicularis muscle without tension, ensuring that it is draped rather than subjected to any traction. Any redundant skin is carefully excised to achieve an optimal aesthetic outcome, while minimizing the risk of complications. Normally 2-3 mm of excess skin is sufficient to avoid any eyelid margin displacement. In other words, if at the end of the operation one finds, for example, 15 mm of excess skin, it will be prudent to remove only 12 mm of it. Please refer to Fig. 1 and Video 1 for more details regarding skin excision. The lateral incision is closed with a running suture using 6/0 Nylon (Ethicon®), while interrupted 6/0 Silk sutures (Ethicon®) are placed along the sub-ciliary region in order to easily squeeze out possible small blood collections.

#### Dressings and Postoperative Management

**Fig. 2:**
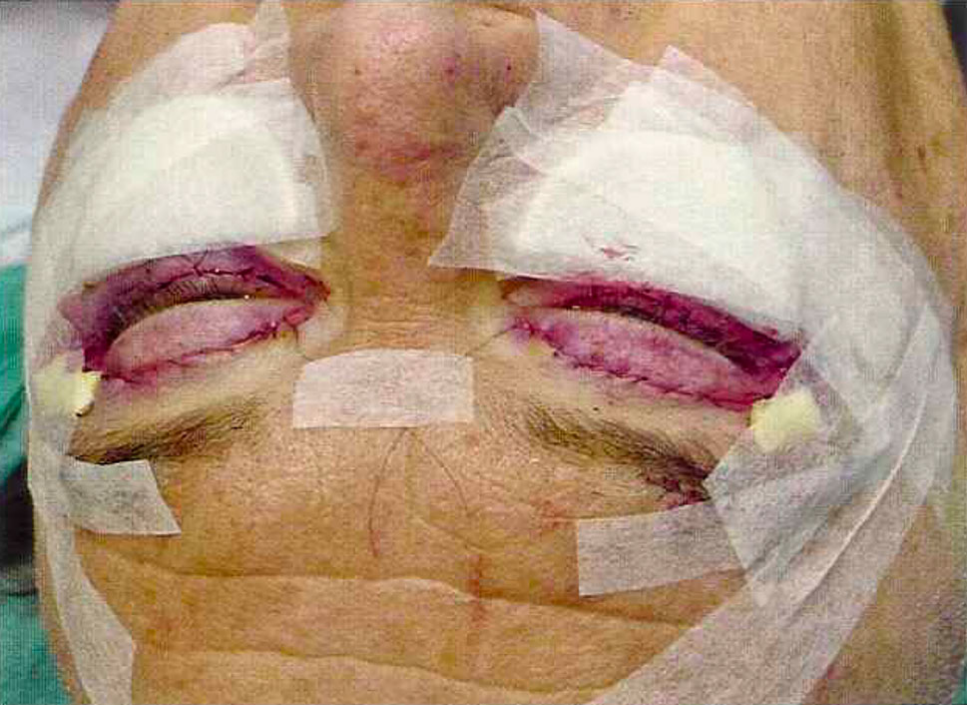
Picture showing the postoperative dressing, left in place for 4 days

Finally, a moderately compressive dressing is applied to support the replaced tissues during the initial postoperative phase. This consists of shaped flat sponges secured with paper adhesive tape and is maintained in place for 4 days, until suture removal (Fig. 2). Tarsorrhaphy is not routinely performed in all patients, but is selectively used in the presence of early signs of conjunctival edema (chemosis), defined as swelling of the conjunctiva with a gelatinous or edematous appearance. In such cases, a lateral temporary tarsorrhaphy is performed approximating the two tarsal plates using two simple interrupted 5/0 silk sutures (Ethicon®), placed approximately 4 and 8 mm from the lateral canthus. The eyelid margins are not freshened, and a central palpebral opening is maintained to allow some visual function. The sutures are usually removed after 7 days. All skin sutures are typically removed between the third and fourth postoperative day. The standard postoperative regimen includes oral cefixime 400 mg once daily for 5 days as antibiotic prophylaxis, paracetamol 1 g as needed for pain control and topical diclofenac sodium eye drops twice daily. Additionally, antibiotic eye drops containing chloramphenicol and betamethasone are prescribed twice daily, and an ophthalmic ointment with tobramycin is applied before bedtime for the first postoperative week.

Video 2 is showing the entire surgical technique.

## Results

**Fig. 3:**
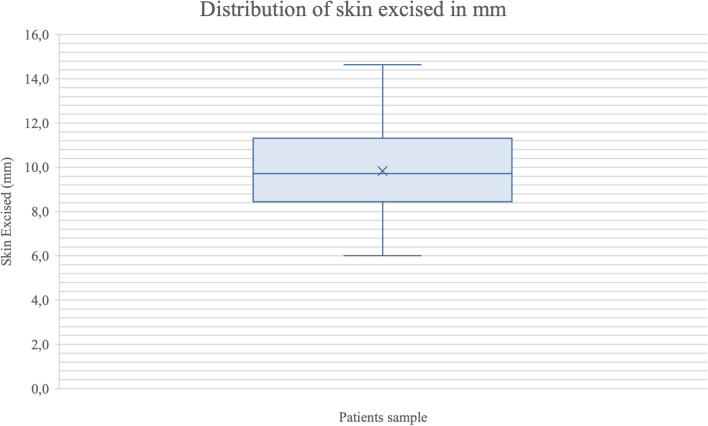
Distribution of the range of skin excised in patients (mm). The box plot displays the median, quartiles and any outliers of the amount of skin removed.

The study population consisted of 93 patients (78 females and 15 males), with a mean age of 58 ± 6.2 years. The amount of skin excised ranged from 6 to 16 mm, with a mean of 10 ± 2.5 mm. All patients were evaluated at 12 months postoperatively. The full study population with demographic data is present in Table 1. To better represent the distribution and variability of the amount of skin excised, a box plot (Fig. 3) was added. The graph shows a median of approximately 9.7 mm. The box plot further indicates that 50% of the procedures involved excising between 8.5 mm (lower quartile) and 11.2 mm (upper quartile) of skin, with the overall range of measurements extending from approximately 6 mm to 16 mm.

**Table 1:** Demographic data

Variable	Value
Number of patients	93
Mean age (± SD)	58 ± 6.2 years
Gender distribution	78 Female (84%)/15 Male (16%)
Mean amount of skin excised (± SD)	10 ± 2.5 mm
Range of skin excised	6–16 mm
Mean follow-up duration	12 months

The follow-up period extended to a final evaluation at one year postoperatively. Patients were examined at regular intervals: 4 days (for dressing and suture removal), 2 weeks, 3 months and 1 year after surgery. All patients enrolled in the study returned for the 1 year follow-up, enabling a comprehensive assessment of aesthetic and functional outcomes. The pre- and postoperative outcomes were evaluated by the senior author, who conducted both clinical and aesthetic assessments. Subjective evaluation was based on both the surgeon's assessment and patient-reported outcomes during follow-up visits. Postoperative photographs were taken following the same photographic protocol as the preoperative images, with the patient positioned at the same distance from the camera (2 meters), in front of a neutral background and with the head in natural position. The same camera model was used for all photographs (Sony NEX-7, lens 28–70 mm, built-in flash), mounted on a tripod to ensure consistency, to ensure comparability between the pre- and postoperative assessments. Videos were recorded using a Panasonic HC-X1200 camcorder.

Regarding complications, one case of ectropion was observed which was successfully managed with a dynamic canthoplasty, resulting in full resolution. Temporary scleral show was recorded in four patients; these cases resolved spontaneously with conservative management, including physiotherapy techniques such as massage and targeted eyelid exercises. One additional patient experienced persistent scleral show, necessitating surgical revision. Three patients developed chemosis, which was treated with tarsorrhaphy sutures took in place for 4 days. All complications are listed in Table 2.

**Table 2:** Complications and management

Complications	N.	Therapeutic approach
Ectropion	1 (1.08%)	Dynamic cathoplasty
Temporary scleral show	4 (4.30%)	Conservative management (massage and exercises)
Persistent scleral show	1 (1.08%)	Surgical revision
Chemosis	3 (3.23%)	Tarsorraphy
Total of complications	9 (9.68%)	

**Fig. 4:**
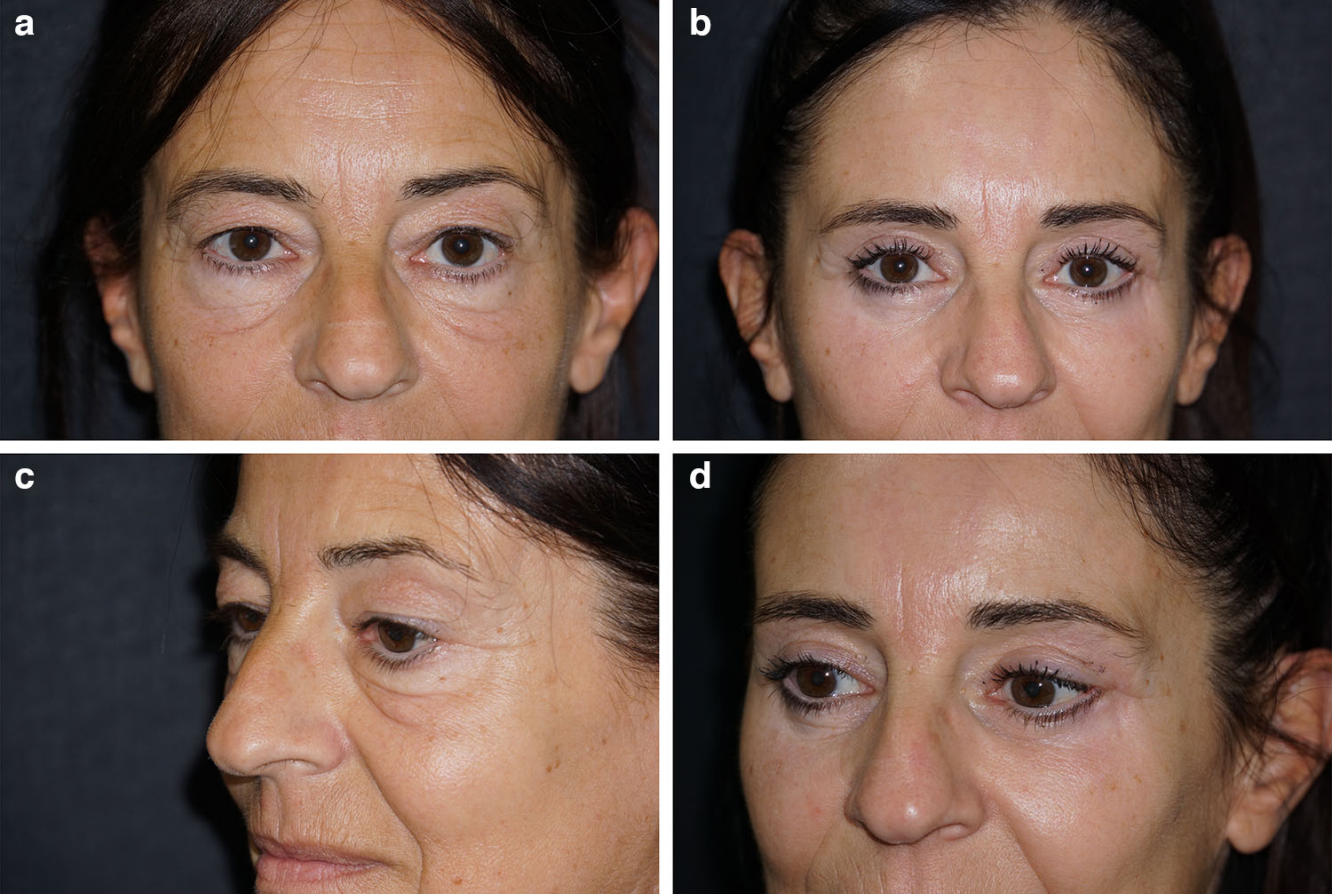
46 y.o. female. **a** preoperative frontal view; **b** postoperative frontal view at 1 year; **c** preoperative left ¾ view; **d** postoperative left ¾ view at 1 year

**Fig. 5:**
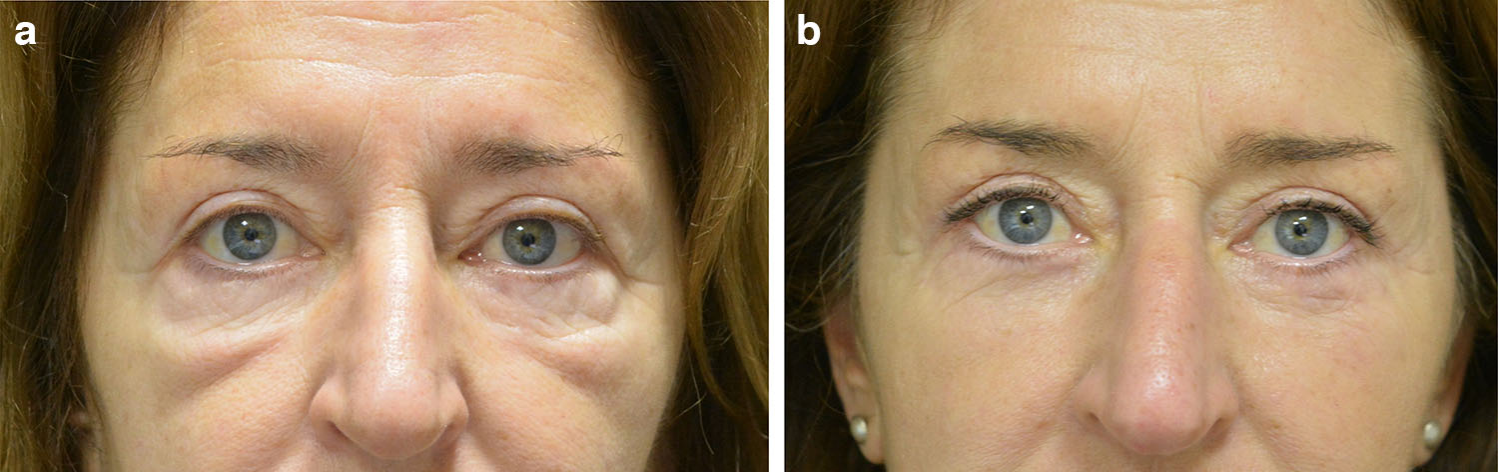
57 y.o. female showing important malar bags bilaterally. **a** Preoperative frontal view; **b** postoperative frontal view at 1 year

**Fig. 6:**
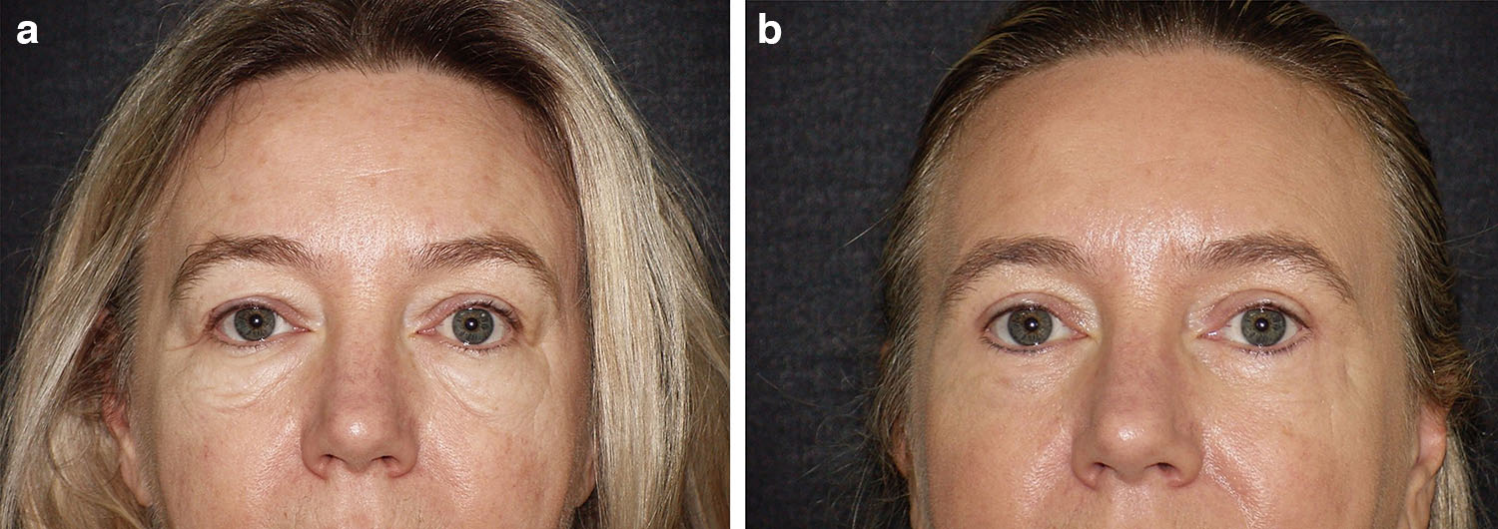
59 y.o. female. **a** Preoperative frontal view; **b** postoperative frontal view at 1 year

Please refer to Figs. 4, 5 and 6 for postoperative outcomes.

## Discussion

The decision to perform an extended lower blepharoplasty rather than a regular “traditional” approach is based on specific indications. Extended blepharoplasty is particularly suitable for patients presenting with festoons, whether composed of skin, orbicularis muscle, fat or a combination of these elements, as well as for those with malar bags. However, in cases where malar bags are primarily due to localized edema, such as with lymphatic stasis, surgical intervention may yield limited or no improvement. A careful preoperative assessment is therefore crucial to determine the underlying cause and guide the surgical plan accordingly. Additionally, the extended lower blepharoplasty might be utilized in those patients presenting with severe lower eyelid and periorbital skin laxity, and infrapalpebral depression. This latter results from a combination of tissue laxity and mild-to-moderate volume loss. In these cases, skin excision is appropriate to correct the excess, while fat grafting may be suitable to correct the volume loss.

In the literature, the term *extended inferior blepharoplasty* is traditionally understood as a midface lift involving an extensive subperiosteal dissection extending to the malar region, with the aim of repositioning the sub-palpebral tissues. [17, 18] The subperiosteal midface lift involves lifting all the soft tissue in the region, from the skin down to and including the periosteum, which must then be incised and released at the base of the dissection because it is inextensible. This operation has specific indications but is of little use if more superficial problems are to be corrected.

We instead define *extended inferior blepharoplasty* as a transcutaneous lower blepharoplasty incorporating both subcutaneous and sub-orbicularis dissection extended about 10 mm or more below the orbital rim. This is followed by meticulous tissue repositioning to achieve a lifting effect in the lower eyelid and in the midface regions. The procedure is further complemented by a robust canthoplasty, either static or dynamic, customized according to the patient’s anatomical characteristics and by the Adamson flap, which ensures stable fixation of the orbicularis oculi muscle.

Several key structures contribute to maintaining the correct position of the lower eyelid margin. The lateral canthal ligament and tendon play a fundamental role in eyelid stability, and their reinforcement and/or repositioning through canthoplasty provides essential structural support. The orbicularis oculi muscle, despite undergoing partial dissection and excision, is repositioned and securely fixed using the Adamson flap, thereby preserving its tone and function. Additionally, the zygomatico-maxillary bony framework and malar fat pads act as a crucial support system for the lower eyelid and for that reason, in case of “negative vector” they should be replenished.

Unlike the “conventional” extended lower blepharoplasty, which involves subperiosteal dissection, the technique we describe entails a more superficial dissection, first between orbicularis and septum and, below the orbital rim, between orbicularis and SOOF.

This technique requires reinforcing the supporting structures of the lower eyelid, thereby reducing the risk of lower eyelid malposition due to compromised support and allows for the removal of a greater amount of excess skin compared to conventional lower blepharoplasty, without major complications. The aim of the article is indeed that of demonstrate that large amount of skin can be removed, as long as the suspension system is stable and effective. Conversely, if excess skin is not adequately removed, the aesthetic result may be suboptimal. However, over-resection must be avoided to prevent excessive tension on the closure, which could lead to complications such as ectropion or eyelid retraction. For this reason, the remaining skin should lie smoothly on the underlying tissues, allowing for a tension-free closure. To this purpose, a small amount of excess skin should be preserved, as the undermined skin over the orbicularis tends to retract slightly postoperatively.

The ability to excise up to 16 mm of skin while maintaining eyelid stability suggests that the extended technique is particularly effective in addressing severe skin redundancy, festoons, and malar bags.

It is also important to highlight the low complication rate observed despite the large series of patients. Only one case of ectropion occurred, which may have resulted from excessive skin resection, although the amount excised was within the average range used in our series. Such complications can also occur with conventional lower blepharoplasty techniques. Notably, despite performing larger-than-usual skin resections in most of the 93 patients, only one case of ectropion required surgical correction. Temporary scleral show was observed in four patients and resolved spontaneously with conservative management, including physiotherapy techniques such as massage and targeted eyelid exercises. However, an annoying complication of this operation can be chemosis. To prevent this complication, at the earliest sign of its potential development, a pair of lateral tarsorrhaphy sutures should be placed and maintained for at least four days. It is also helpful to prescribe the patient hypertonic eye drops alternating with steroid eye drops. In these cases, corticosteroids are also given orally for a few days. Overall, the majority of patients achieved satisfactory aesthetic and functional outcomes with no significant long-term complications.

Although in the presence of clear atrophy of the adipose layer we also attempt to restore it by grafting an adequate amount of fat, this study specifically focused on a selected cohort of patients in whom the main clinical findings were cutaneous elastosis and soft tissue laxity, with little to no evidence of adipose tissue depletion. In such cases, volume restoration was not a primary concern, and the treatment strategy was based on skin excision and soft tissue repositioning. We agree that fat grafting has become a valuable tool in midface rejuvenation, particularly in patients experiencing volume loss in the malar and infraorbital areas. Likewise, we acknowledge that age-related bone resorption contributes significantly to midfacial descent by weakening structural support. In clinical practice, when skeletal atrophy is the predominant issue, especially in patients with a negative vector, we recommend volume restoration through malar implants, such as extended submalar designs (e.g., flowers-type), to restore projection and support. However, to objectively assess the efficacy of the extended blepharoplasty technique alone, cases treated with implants or fat grafting were intentionally excluded from this study. It is important to note that, when correctly performed, soft tissue repositioning offers stable and predictable long-term outcomes. In contrast, the behavior of grafted fat remains less reliable, with a known risk of partial or complete resorption in a subset of patients.

Despite these promising findings, this study has some limitations. It is a single-center study, which may limit the generalizability of our results. Furthermore, the absence of a FACE-Q evaluation before and after surgery prevents a standardized assessment of patient-reported outcomes. However, we believe the study remains significant, as it demonstrates that extended lower blepharoplasty allows, when necessary, for the removal of substantial amounts of skin while maintaining a low complication rate. Future studies incorporating patient-reported outcome measures and multi-center data would further validate these findings.

In order to avoid confusion, we would like to clarify that we do not agree with the classification proposed by some, according to which canthopexy would be canthal ligament reinforcement without canthotomy, whereas canthoplasty should be understood as anchoring the canthal ligament to the orbital rim after canthotomy. The etymology of these words of Greek origin suggests quite different meanings, and thus, in our opinion, canthoplasty should include any surgery performed in the canthal area.

## Conclusion

The extended lower blepharoplasty technique is a safe and effective approach for patients with redundant skin laxity in the lower eyelid, festoons and malar bags, allowing for substantial skin resection without compromising lower eyelid stability. The low rate of complications and the favorable aesthetic and functional outcomes suggest that this technique is a viable alternative to “traditional” subperiosteal midface lift, which still maintains a consistent set of indications. While the study is limited by its single-center design and the lack of standardized patient-reported outcomes, the findings remain clinically relevant and encourage further research to confirm the benefits of extended lower blepharoplasty on a larger scale.

## Supplementary Information

Below is the link to the electronic supplementary material.

Supplementary file1 (MP4 39470 KB)

Supplementary file2 (MP4 76414 KB)
